# Unravelling effects of relative humidity on lipid barrier formation in human skin equivalents

**DOI:** 10.1007/s00403-019-01948-3

**Published:** 2019-07-18

**Authors:** Arnout Mieremet, Walter Boiten, Rianne van Dijk, Gert Gooris, Herman S. Overkleeft, Johannes M. F. G. Aerts, Joke A. Bouwstra, Abdoelwaheb El Ghalbzouri

**Affiliations:** 1 Department of Dermatology, Leiden University Medical Center, Albinusdreef 2, Room C3-76, 2333 ZA Leiden, The Netherlands; 2 Research Division BioTherapeutics, Leiden Academic Centre for Drug Research, Leiden University, Leiden, The Netherlands; 3 Department of Bio-organic Synthesis, Leiden Institute of Chemistry, Leiden University, Leiden, The Netherlands; 4 Medical Biochemistry, Leiden Institute of Chemistry, Leiden University, Leiden, The Netherlands

**Keywords:** Artificial skin, Humidity, Cell-culture techniques, Ceramides, Molecular probes

## Abstract

Relative humidity (RH) levels vary continuously in vivo, although during in vitro generation of three-dimensional human skin equivalents (HSEs) these remain high (90–95%) to prevent evaporation of the cell-culture medium. However, skin functionality is directly influenced by environmental RH. As the barrier formation in HSEs is different, there is a need to better understand the role of cell-culture conditions during the generation of HSEs. In this study, we aim to investigate the effects of RH on epidermal morphogenesis and lipid barrier formation in HSEs. Therefore, two types of HSEs were developed at 90% or at 60% RH. Assessments were performed to determine epidermal morphogenesis by immunohistochemical analyses, ceramide composition by lipidomic analysis, and lipid organization by Fourier transform infrared spectroscopy and small-angle X-ray diffraction. We show that reduction of RH mainly affected the uppermost viable epidermal layers in the HSEs, including an enlargement of the granular cells and induction of epidermal cell activation. Neither the composition nor the organization of the lipids in the intercorneocyte space were substantially altered at reduced RH. In addition, lipid processing from glucosylceramides to ceramides was not affected by reduced RH in HSEs as shown by enzyme expression, enzyme activity, and substrate-to-product ratio. Our results demonstrate that RH directly influences epidermal morphogenesis, albeit the in vitro lipid barrier formation is comparable at 90% and 60% RH.

**Electronic supplementary material:**

The online version of this article (10.1007/s00403-019-01948-3) contains supplementary material, which is available to authorized users.

## Introduction

Main functions of the skin are to protect the body from water loss and against exogenous factors, which are primarily performed by the stratum corneum (SC). Skin functionality is influenced by environmental relative humidity (RH), which ranges worldwide from 5–99% RH with a median of 70–80% RH over land areas [10]. The RH affects susceptibility to mechanical stress and fracture, elasticity, protein organization, lipid conformation, and epidermal hydration [4, 8, 15, 18, 34, 42, 55]. In vivo studies revealed that high RH levels delayed epidermal barrier repair and reduced the level of natural moisturizing factor [15, 18]. Furthermore, switching from high to low RH affected total epidermal water loss (TEWL) values, a widely applied analysis to determine the skin barrier function [37, 23, 26]. This indicates the adaptation of the skin barrier formation to fluctuating RH levels [45].

Despite the interactions between RH and the skin, during the generation of three-dimensional human skin equivalents (HSEs), high RH levels (90–95%) are used as the standard cell-culture condition. This difference between in vivo and in vitro RH levels could contribute to the altered barrier formation of HSEs compared to that of native human skin (NHS) [13, 36, 38, 48]. Only a limited amount of comparative studies addressed the influence of RH levels in vitro [6, 7, 28, 46, 47]. These showed that epidermal morphogenesis was affected by a reduction in RH, albeit not uniformly in all HSE types. Moreover, an improved skin hydration was observed, at which 60% RH was reported to be an optimal in vitro level [6]. Furthermore, the HSEs developed at reduced RH were shown to have enhanced barrier properties [46]. Nevertheless, it remains unresolved by which mechanism the epidermal barrier is reinforced.

The SC consists of fully keratinized corneocytes surrounded by a lipid matrix, which is the only continuous pathway through the SC [14, 40]. Therefore, the lipid matrix is highly important for the functionality of the epidermal barrier. It consists of a specific lipid composition (i.e., ceramides, cholesterol, and free fatty acids), assembled in a characteristic lipid organization (i.e., lateral and lamellar). The 12 most common ceramide subclasses were analysed in this study, referred to as total ceramides (CERs_total_). These were subcategorized in ω-esterified ceramide (CERs EO) and in (non-ω-esterified) ceramide (CERs) subclasses [25, 53, 54]. The previous studies revealed a different composition of CERs_total_ and an altered lipid composition indicative for the altered barrier formation in HSEs as compared to NHS [48, 50].

The interaction between RH and the lipid barrier formation in HSEs has been studied in submerged (> 100% RH) and in reduced RH (< 90% RH) conditions. When developed at submerged conditions (> 100% RH), the level of ceramide precursor (acyl)glucosylceramide was elevated, leading to a lower level of ceramide EOS as compared to air-exposed cultures (93%) [49]. This could be a consequence of elevated pH values at submerged or occluded conditions [42], which may affect the hydrolytic activities of β-glucocerebrosidase-1 (GBA) or acid sphingomyelinase (aSMASE). These enzymes convert glucosylceramide and sphingomyelin, respectively, to ceramide at the stratum granulosum (SG)–SC interface [16, 52]. Opposite to submerged conditions, in HSEs generated at reduced RH (50%), the expression of glucosylceramide synthase was upregulated [46], indicating that RH is associated with biosynthesis and processing of glucosylceramides. Nevertheless, it remains to be determined whether RH levels directly affect lipid composition and organization in the SC of in vitro developed HSEs.

In this study, we aim to determine if RH levels affect epidermal morphogenesis and barrier lipid formation in HSEs. Therefore, full-thickness models (FTMs) and collagen–chitosan full-thickness models (CC-FTMs) were generated at 90% and 60% RH. Both HSE types encompass the epidermal and dermal equivalents, although the CC-FTM has a strengthened dermal extracellular matrix due to the added biopolymer chitosan and improved barrier formation [30]. In addition, we examined the conversion of precursor glucosylceramide to ceramide at the SG–SC interface to obtain more detailed information on this process at various RH levels.

## Materials and methods

### Generation of FTMs and CC-FTM at reduced RH

Declaration of Helsinki principles is followed during the obtainment of primary cells from surplus female adult mama skin tissue, as stated earlier [21, 29]. Isolation of primary cells from the dermis and epidermis and cell culturing was performed as described before [12, 51]. All isolated primary cells were tested and found negative for mycoplasma contamination. FTMs and CC-FTMs were generated as described before [30, 39, 48]. The reduction in RH was performed from 90% gradually in steps of 7.5% each day initiated after 7 day air exposure to 60% using a Memmert INC153med CO_2_ incubator with humidity module (Memmert, Schwabach, Germany). Medium was refreshed every other day after 7 day air exposure. HSEs were developed for a total of 16 day air-exposed. HSE batches were generated with four unique primary cell donors.

### Immunohistochemical analyses

Sections of HSEs or NHS were formaldehyde fixated and embedded in paraffin or snap frozen in liquid nitrogen [31]. Haematoxylin and eosin (HE) staining was performed according to manufacturer’s instructions (Klinipath, Duiven, The Netherlands). Protein analyses by immunohistochemistry or indirect immunofluorescence were performed on 5 μm sliced paraffin embedded cross sections. After deparaffinization and rehydration, heat-mediated antigen retrieval in citrate buffer pH 6 occurred. Antigen retrieval for collagen type IV staining was mediated by protease incubation. Non-specific antibody binding was reduced through blocking with normal human serum (Sanquin, Leiden, The Netherlands). Immunohistochemical analyses were performed using the streptavidin–biotin–peroxidase system (GE Healthcare, Buckinghamshire, United Kingdom) according to the manufacturer’s instructions or using indirect immunofluorescence, as described before [30]. Specifications of the antibodies are provided in supplementary Table 1. Control immunostainings revealed no unspecific binding or background staining of the secondary antibody (Supplementary Fig. S1a). Number of corneocyte layers was determined by safranin red staining and potassium hydroxide expansion of the SC [43, 44]. Estimations of the epidermal thickness and the proliferation index were performed as reported earlier [1, 30]. Activity-based probe assay using MDW941 was performed as reported earlier by van Smeden et al*.* [52].

### Stratum corneum lipid composition

Extraction of SC lipids was performed based on an adjusted Bligh and Dyer method as described by Boiten et al*.* [2]. The dry SC was weighed before and after lipid extraction using a microbalance. The lipids were analysed by liquid chromatography coupled to mass spectrometry (LC–MS) according to the methods as described elsewhere [2]. Details of instrument settings and data quantification are described in supplementary materials and methods.

### Stratum corneum lipid organization

Determination of lamellar organization was performed by small-angle X-ray diffraction [19] and determination of lateral organization was performed by Fourier transform infrared spectroscopy (FTIR) [32, 33], according to methods described in supplementary materials and methods.

### Statistics

Statistical analyses are conducted using GraphPad Prism version 7.00 for Windows (GraphPad Software, La Jolla California USA). Statistical testing was performed with one-way or two-way ANOVA with Tukey's post-test. Significance is shown for comparison between high and low RH (with lines) within an HSE type and for comparison between all HSEs versus NHS (above NHS), otherwise specifically stated. No comparisons between FTMs and CC-FTMs were performed due to primary donor effect. Statistical differences are noted as *, ** or ***, corresponding to *P* < 0.05, < 0.01, < 0.001.

## Results

### Epidermal morphogenesis in HSEs developed at reduced relative humidity

**Fig. 1:**
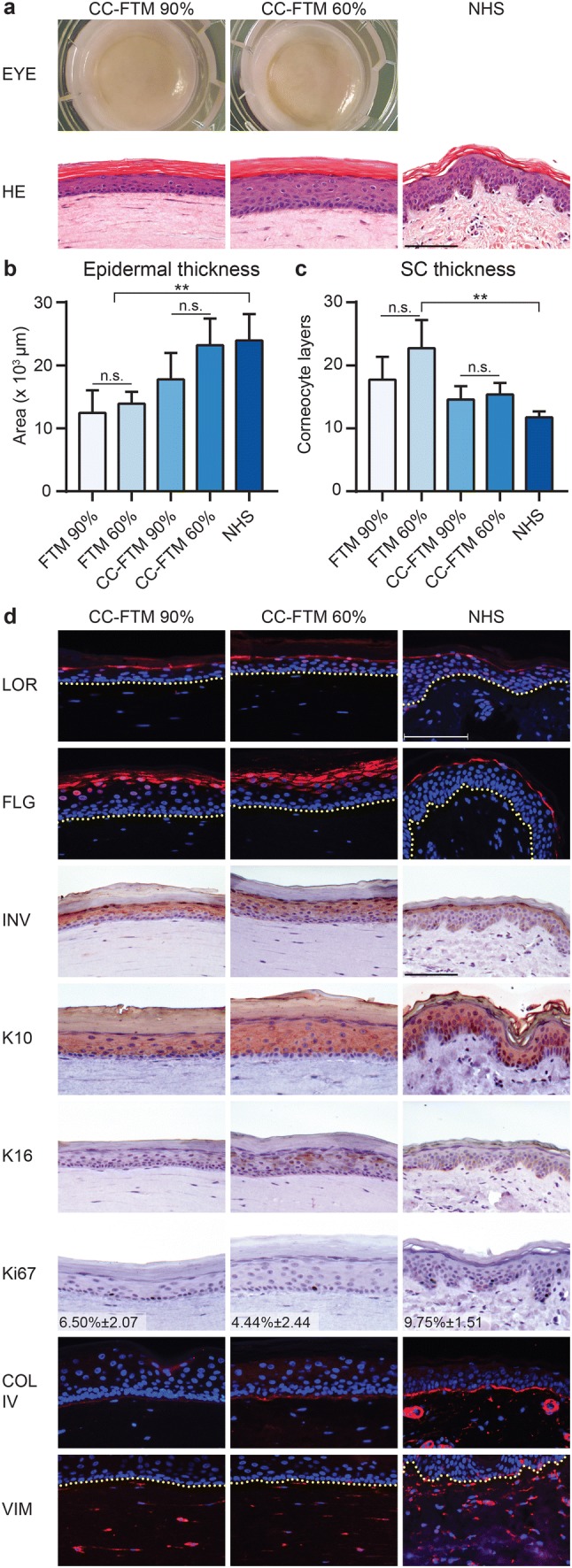
Generation of HSEs at reduced relative humidity. **a** General morphology of CC-FTMs generated at high (90%) and low (60%) RH, assessed by eye or after HE staining. **b** Epidermal thickness of HSEs and of NHS, indicated by mean + SD, *N* = 4. **c** Number of corneocyte layers in HSEs and in NHS. Data indicates mean + SD, *N* = 4. **d** Epidermal morphogenesis, basement membrane formation, and fibroblast distribution in CC-FTMs and NHS. Expression of loricrin (LOR), filaggrin (FLG), involucrin (INV), keratin 10 (K10), and keratin 16 (K16) indicates epidermal differentiation programs or activation. Proliferation was assessed by Ki67 (with indicated proliferation index as mean ± SD, *N* = 4), basement membrane formation by collagen type IV (COL IV), and fibroblast distribution by vimentin (VIM) expression. Protein biomarkers are shown in red and nuclei in blue. Yellow dotted line indicates dermal–epidermal junction. Scale bar indicates 100 μm

To thoroughly and robustly assess the effect of RH in HSEs, we generated FTMs and CC-FTMs at 90% and 60% RH. The reduction in RH was performed gradually after the first SC layers were formed to protect the viable epidermis from excessive desiccation [6]. Assessments of macroscopic appearance and general morphology were performed for CC-FTMs and NHS (Fig. 1a) and for FTMs (Supplementary Fig. S2a). The epidermis contained four distinguishable epidermal layers in both HSE types, irrespective of RH levels. In addition, the thickness of the viable epidermis and number of corneocyte cell layers in the SC was similar at 90% and 60% RH in both HSE types (Fig. 1b, c). In HSEs generated for 16 days, the number of corneocyte layers was slightly higher than that of NHS. Noteworthy, the corneocytes of both HSE types expanded more in the alkali environment that the corneocytes of NHS (Supplementary Fig. S1b).

The expression of protein biomarkers in HSEs and NHS was evaluated to assess the effect of RH on epidermal morphogenesis, basement membrane (BM) formation, and fibroblast distribution in CC-FTMs and NHS (Fig. 1d), and in FTMs (Supplementary Fig. S2b). The epidermal terminal and late differentiation programs were examined by expression of loricrin, filaggrin, and involucrin. This revealed an increased thickness of the SG in CC-FTMs generated at 60% RH, but not in FTMs at 60% RH. Involucrin was also expressed in the spinous layer of HSEs, in contrast to its restricted expression in the SG of NHS. Similar execution of the early differentiation program was detected in all conditions based on the suprabasal keratin 10 expression. Epidermal activation was increased at reduced RH in CC-FTMs based on the expression of keratin 16 (Fig. 1d). Elevated epidermal activation could not be detected in FTMs, due to the expression of keratin 16 at 90% RH. In all HSEs and NHS, the expression of keratin 17 was absent, indicating no severe epidermal activation induced by reduced RH (Supplementary Fig. S2c). The cell proliferation marker Ki67 remained located at the basal layer in all conditions, with comparable proliferation indexes at high and low RH. The expression of collagen type IV was unaffected by the reduced RH, indicating similar BM formation. Furthermore, the distribution of the fibroblasts was analysed by vimentin expression, which was similar at both RH levels. As compared to NHS, the deposition of the BM was reduced and the distribution of the fibroblasts was homogeneous. Altogether, these results indicate that reduction in RH mainly affected the uppermost layers of the epidermis.

### Lipidomic profiling of stratum corneum ceramides

**Fig. 2:**
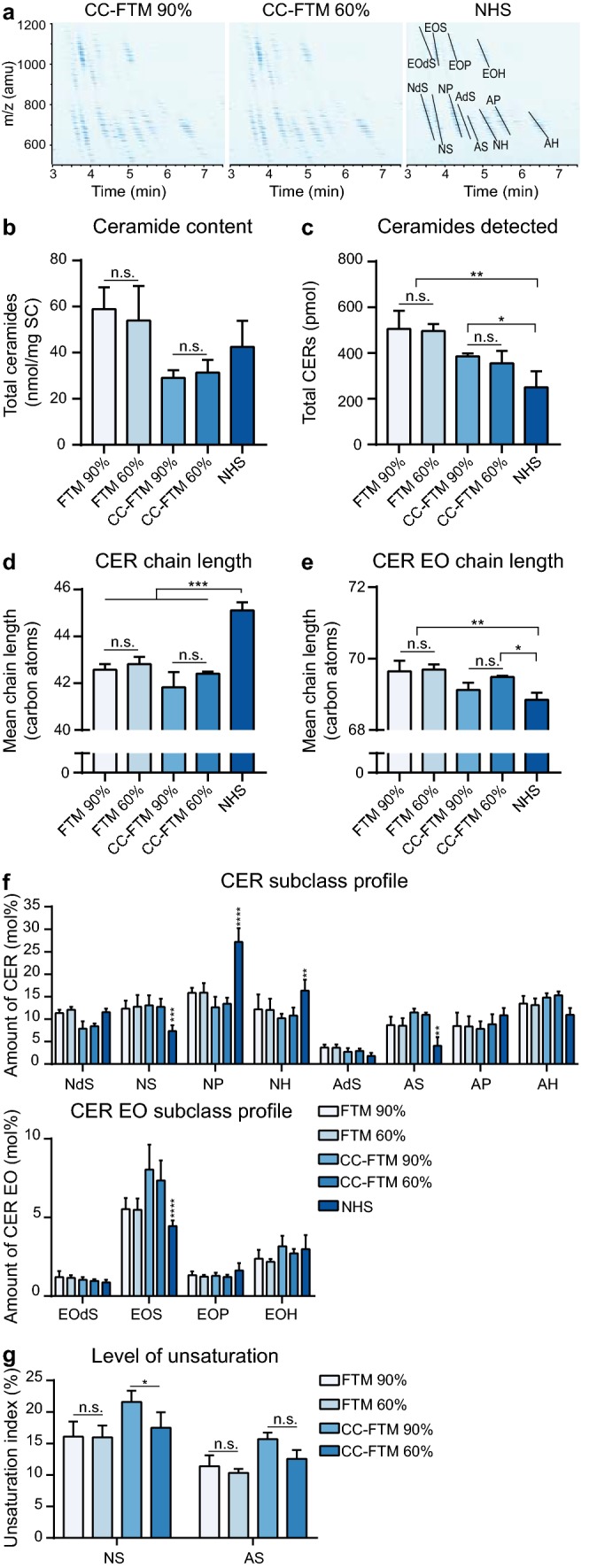
Stratum corneum ceramide composition of HSEs generated at reduced relative humidity. **a** Representative ion maps of CC-FTMs generated at 90% and 60% RH and of NHS. Twelve CERs_total_ subclasses are indicated by position in the ion map of NHS and are named according to Motta et al*.* [35]. Ion maps are shown as time after elution on the *x*-axis and mass in atomic mass unit (amu) on the *y*-axis. **b** Level of CERs_total_ ceramides in the SC per mg SC of indicated HSEs and of NHS. **c** Total amount of CERs in 1500 ng lipid extract as detected by LC–MS. **d** Bar diagram plot of the mean carbon chain length of CERs. **e** Bar diagram plot showing the CERs EO mean carbon chain length. **f** CER and CER EO subclass profiles in relative amount. **g** Bar diagram plot of the unsaturation index in CER subclasses NS and AS of indicated HSEs. All data represent mean + SD, *N* = 4

To gain more information on the barrier formation, the ceramide composition was investigated using an advanced liquid chromatography coupled to mass spectrometry (LC–MS) analysis. All CERs_total_ subclasses were detected in both HSE types and in NHS on the LC–MS ion maps (Fig. 2a). An increased presence of low mass CERs was detected (below 600 atomic mass units) in HSEs compared to NHS. Quantification of the detected signal was performed to determine the level of CERs_total_ per mg SC. This revealed no significant change in the absolute amount of CERs_total_ after generating HSEs at reduced RH compared to high RH (Fig. 2b). In addition, a comparison between the cumulative detected CERs_total_ per injection in the LC–MS revealed similarity in HSEs generated at high and low RH (Fig. 2c). The mean carbon chain length (MCL) of CERs and CERs EO was calculated, which revealed no significant difference for neither the MCL of CERs nor for the MCL of CERs EO between high and low RH (Fig. 2d, e). The MCL of CERs in NHS was significantly higher as compared to HSEs, whereas the MCL of CERs EO in NHS was more comparable to that of HSEs. More detailed information on the chain length of CERs and CERs EO was obtained by plotting the distribution by carbon atom number, for even numbered CERs ranging from C32 (32 carbon atoms) to C54 and for CERs EO ranging from C64 to C74 (Supplementary Fig. S3a–d). This revealed no substantial differences between high and low RH levels, solely CER EO C68 in CC-FTMs was significantly reduced at 60% RH. Compared to NHS, the fraction of CERs ≤ C42 was increased and the fraction of CERs ≥ C44 was reduced in HSEs in relative amounts. Furthermore, the CERs EO chain length distribution was generally similar in NHS and HSEs in relative and absolute values, except for FTMs as these contain a higher level of CERs EO C70 and C72 per mg SC. Then, the CERs_total_ subclass profiles were determined and compared in HSEs generated at varying RH levels for relative (Fig. 2f) and for absolute amounts (Supplementary Fig. S4). No differences were observed between high and low RH in both comparisons. As compared to NHS, alterations in the subclass profile (i.e., subclasses NS, NP, AS, and EOS) were detected in both HSE types. Considering the absolute amounts, the CC-FTMs resembled NHS to a higher extent in subclass profile, although important deviations persisted. Composition assessment was continued by the determination of the level of unsaturation in the CER subclasses AS and NS. Due to the low abundance of unsaturation of these CERs in NHS, these levels were not further analysed [2]. The percentage of unsaturation (i.e., unsaturation index) was determined in CER subclasses NS and AS (Fig. 2g). At 60% RH, a reduction in the unsaturation index was observed for subclass NS as compared to high RH in CC-FTM, whereas in FTMs, no changes were observed due to altered RH. The unsaturation index for these subclasses provides an indication on the change in level of unsaturation for the other CERs_total_ subclasses.

### Glucosylceramide processing at varying RH levels

**Fig. 3:**
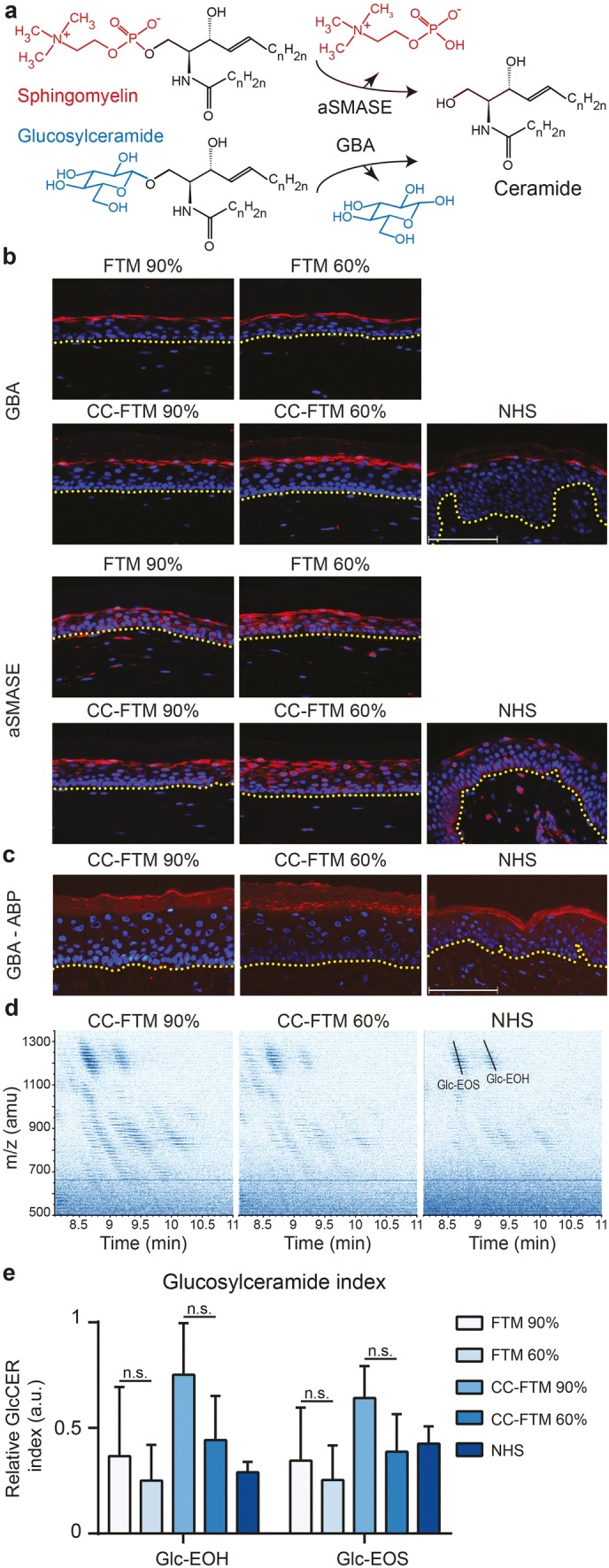
Precursor ceramide conversion in HSEs generated at reduced relative humidity. **a** Illustration of terminal ceramide processing mediated by β-glucocerebrosidase-1 (GBA) and acid sphingomyelinase (aSMASE). **b** Protein expression of GBA and aSMASE in HSEs and NHS. **c** Activity-based probe (ABP) fluorescence signal of MDW941, which solely binds to the active GBA enzyme. Protein expression and ABP signal are shown in red and nuclei are shown in blue, and yellow dotted line indicates dermal–epidermal junction. Scale bar indicates 100 μm. **d** Ion maps of the GlcCERs in CC-FTMs and NHS. **e** Relative values of the glucosylceramide index of CER EO subclasses EOS and EOH. Data represent mean + SD, *N* = 4

We further studied the terminal ceramide processing which occurs at the SG–SC interface. The conversion of precursor ceramides into ceramides is mediated by the enzymes aSMASE and GBA (Fig. 3a). To fully comprehend the effect of RH on this process, we analysed enzyme protein expression, enzyme activity, and substrate-to-product ratio. In both HSE types, GBA expression was detected in the SG and aSMASE expression was highest in the SG, similar as in NHS (Fig. 3b). In CC-FTMs generated at 60% RH, GBA and aSMASE were present in more cell layers of the SG, in line with the results of epidermal morphogenesis. Active GBA was detected in situ using the activity-based probe MDW941, a fluorescent suicide inhibitor. Active GBA molecules resided at the SG–SC interface and this distribution was similar at both RH levels and in NHS (Fig. 3c). The ratio between glucosylceramide (GlcCER) to ceramide was determined by LC–MS. The presence of the higher molecular weight GlcCERs for most of the CER subclasses was detected on the LC–MS ion map (Fig. 3d). We determined the relative GlcCER indexes for subclasses EOS and EOH, which are exclusively processed by GBA [52]. In addition, this provides an indication for the GlcCER index in CERs_total._ Comparing the GlcCER indexes between high and low RH and to NHS, no significant differences were observed in this data set (Fig. 3e).

### Organization of the stratum corneum barrier lipids

**Fig. 4:**
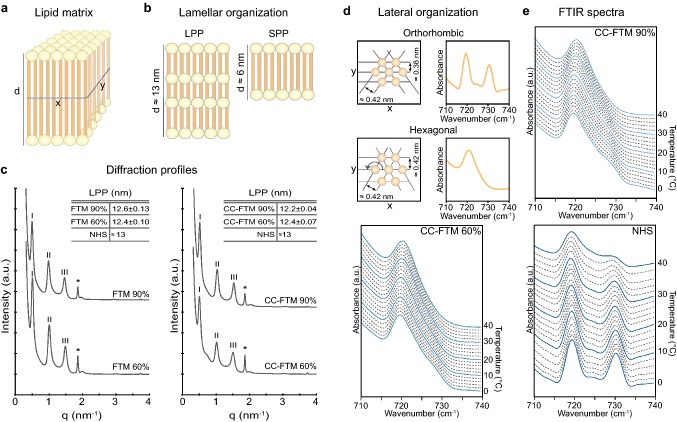
Lipid organization in HSEs generated at reduced relative humidity. **a** Schematic overview of the 3D lipid matrix in the SC. Lipids are shown with head group region in yellow and hydrocarbon chain region in orange. **b** Illustration of the lamellar phases. The long periodicity phase (LPP) has an approximate repetition distance (*d*) of 13 nm and the short periodicity phase (SPP) has an approximate d of 6 nm [5]. **c** Representative X-ray diffraction profiles. Peaks attributed to the LPP are indicated by Roman numbers. The peak of crystalline cholesterol phase is indicated by the asterisk symbol (*). Inset shows the repeat distances of the LPP as mean ± SD, *N* = 4, whereas for NHS it was derived from Bouwstra et al*.* [5]. **d** Schematic overview of lateral lipid organization. Hydrocarbon chains can adopt a very dense orthorhombic or a dense hexagonal packing. This is indicated by a double or single peak, respectively, in the methylene rocking vibration region after measurement by FTIR. **e** Representative FTIR spectra of the methylene rocking vibrational mode of lipids in CC-FTMs and NHS

Analyses of the lipid organization provides additional information on the barrier formation at varying RH levels. The intercorneocyte lipid matrix contains repetitive lamellar stackings (Fig. 4a, b), which were studied by small-angle X-ray diffraction. The diffraction profiles revealed the presence of the long periodicity phase (LPP) at 90% and 60% RH in both HSE types (Fig. 4c). The repeat distance of the LPP was longer in the CC-FTMs generated at reduced RH, whereas in FTMs, this remained equal. As compared to NHS, the LPP repeat distance is shorter and there is no SPP formed in both HSE types [5]. Within the lamellae, lipids are organized in either a very dense orthorhombic or in a dense hexagonal lateral packing (Fig. 4d). The lateral packing was determined in HSEs generated at high and low RH and appeared to be hexagonal in the CC-FTMs (Fig. 4e) as well as in the FTMs (data not shown), irrespective of RH level. In NHS, the lipids were predominantly ordered in the orthorhombic lateral packing.

## Discussion

The objectives of this study were to determine the effects of RH on epidermal morphogenesis and on lipid barrier formation during in vitro development of HSEs. In addition, whether RH affects the conversion of GlcCER into CER was studied. This study shows that the reduction of RH mainly influenced the differentiation in the uppermost epidermal layers and that it resulted in epidermal activation, although variations between HSE types were observed. Strong alterations for neither SC ceramide composition nor lipid organization were observed. In addition, the conversion of GlcCER into CER at the SG–SC interface remained unaffected by variations in RH. By optimization and standardisation of the in vitro cell-culture conditions, more robust and clinically significant HSEs representing healthy and diseased skin will be generated. This makes HSEs a valuable tool in preclinical research.

Our data are in agreement with the diversity in adaptations after RH reduction in different HSE types. The FTMs data showed no effect on epidermal morphogenesis, which also has been observed before [6]. The CC-FTM data showed an enlarged SG at reduced RH, in line with the previous results in epidermal models [7, 46]. More research is required to explain this diversity, although the dermis is likely to play a contributing factor in adaptation to reduced RH. Another morphogenetic adaptation to lower RH was the higher epidermal cell activation, which has been aggravated at levels below 60% RH (J.A. Bouwstra, unpublished results). This could be due to an accelerated differentiation program and barrier formation as response to the lower external water level, inducing temporary but fierce signalling for barrier repair responses [20]. In addition, reduced RH levels affected the activation of dermal fibroblasts, which could contribute to more epidermal activation [56, 57]. As an activated epidermis is shown to undermine the lipid barrier formation in vitro and in vivo [9, 41], lower in vitro RH levels are not recommended.

The variance in lipid composition between high and low RH was small, leading to an unaltered SC lipid organization in HSEs. The differences in lipid composition between HSEs and NHS are related to the differences in lipid organization, which is in line with the previous reports [48, 50]. Although the contribution of each lipid molecule to the matrix organization is not fully elucidated, it is known that major contributors to a reduced repeat distance of the LPP are the disbalanced CER subclass profile in combination with the reduced MCL of CERs [24]. The lack of formation of SPP is ascribed to the overabundance of CER EO subclasses [3, 11]. Moreover, the formation of a hexagonal lateral packing in HSEs is associated with the increased presence of monounsaturated CERs, and the increased presence of CERs with a shorter chain length [24, 50]. These aspects remain important targets for future studies to better mimic the barrier formation of NHS in HSEs.

Novel insights on the effect of RH on the conversion of GlcCER into CERs were obtained. Earlier findings in dry (50% RH) HSEs revealed an upregulated expression of glucosylceramide synthase, indicating an upregulation of total CER synthesis [46], whereas in submerged (> 100% RH) HSEs, an accumulation of GlcCER in the lipid matrix was observed [49]. Our results show similarity in GlcCER indexes, absolute ceramide content, GBA enzyme expression, and GBA activity at high and low RH levels. These complementary findings indicate that ceramide biosynthesis is not affected by reduced RH levels. Although in submerged HSEs there is an accumulation of GlcCERs [49], mediated by increased synthesis or reduced conversion after air-exposure, the terminal ceramide processing is not impeded by high in vitro RH levels.

The expected epidermal hyperplasia in FTMs was not observed in this study [31, 48], most probably caused by primary donor effects or increased rate of medium refreshments, but will not impair our conclusions [30, 31]. Furthermore, due to a high technical complexity in the data analysis, in this study, no unsaturated CERs EO with a linoleic chain (contributing ~ 17–19% to FTM and NHS; Helder et al*.*, in preparation) and saturated CERs EO with an oleic chain (contributing ~ 23% to FTM; Helder et al*.*, in preparation) were quantified. This leads to the underestimation of the abundance of CERs EO. Although FTMs and CC-FTMs were generated with different primary cell donors limiting a direct comparison between both, it appears reasonable that the morphogenesis and barrier formation in CC-FTMs better resemble that of NHS in line with the previous findings [30]. An overall limitation of both HSE types is the lack sweat and sebum production, of which the salt, glycerol, and lactate are known to affect the hydration status and barrier functionality of the SC [6, 17, 22, 27]. These factors should also be considered in future studies regarding the interplay between RH and HSEs.

This study revealed that the reduction of external relative humidity levels during in vitro development of human skin equivalents does not lead to strong alterations in the epidermal lipid barrier formation. This study contributes with novel insights to a better understanding of the influence of cell-culture conditions on the lipid barrier formation in HSEs.

## Electronic supplementary material

Below is the link to the electronic supplementary material.

Supplementary file1 (DOCX 986 kb)
