# Supplementary material for: Unravelling effects of relative humidity on lipid barrier formation in human skin equivalents
Source: Arch Dermatol Res. 2019 Jul 18;311(9):679–89. doi: 10.1007/s00403-019-01948-3 (PMC6787114; doi:10.1007/s00403-019-01948-3)
Supplement: Supplementary file 1 — Supplementary file1 (DOCX 986 kb) [file 403_2019_1948_MOESM1_ESM.docx]

# Supporting Information

**Supplementary Materials and Methods**

**LC-MS settings and data quantification**

The LC-MS methods were used as described before by Boiten *et al.* [[2](#_ENREF_2)]. SC lipid extracts concentration was set at 0.3 mg/ml of which 5 μl was injected. Samples were separated on a PVA-Sil column (5 μM particles, 100x2.1 mm i.d.)(YMC, Kyoto, Japan) with an Acquity UPLC H-class (Waters, Milford, MA, USA). CERs_total_ were detected using a XEVO TQ-S mass spectrometer (Waters, Milford, MA, USA). Measurements were performed in full scan mode from 1.25-8.00 minutes between m/z 350–1200 and from 8.00-12.5 minutes between m/z 500-1350 [[32](#_ENREF_32)]. The area under the curve (AUC) of a chromatogram from the mono-isotopic mass of the main fragment of each ceramide was integrated. For a selected number of CERs, the relative abundance overlaps of the corresponding monounsaturated CERs containing two naturally abundant ^13^C was more than 10%. When this occurred, the AUC of the monounsaturated CERs containing two naturally abundant ^13^C was determined. Using its natural isotope distribution, the overlap was calculated and subtracted from the one of with which it overlapped for correction. Quantification to the molar amount of CERs_total_ occurred using the AUC of the chromatogram of any specific ceramide, the internal standard of CER N(24deuterated)S(18) and a 3D response model based on compound properties and a calibration curve from a limited number of synthetic ceramides (**Supplementary Fig. S5**) [[29](#_ENREF_29)]. Quantified values of the glucosylceramide index of subclasses EOS and EOH are determined by dividing the AUC values of total GlcCER signal by that of cumulative AUC values of corresponding CER EO, as described by van Smeden [[52](#_ENREF_52)]. As this data is not quantified, it is presented as arbitrary amount.

**Small angle X-ray diffraction**

Measurements were performed at the European Synchrotron Radiation Facility at station BM26B as described in detail by Mojumdar *et al.* [[33](#_ENREF_33)]. Measurements occurred twice during 90 seconds to overcome a gap in signal detection, with a sample distance to pilatus1M detector of 2.1 meter. Calibration methods and data conversion from x,y coordinates to polar coordinates were performed as described by Groen *et al.* [[19](#_ENREF_19)]. From the positions of a series of equidistant diffraction peaks, the repeat distance (d) of a lamellar phase was calculated using the equation d = n ∙ 2π / q_n_, where q is the scattering vector and n is the order number of the diffraction peak.

**Fourier transform infrared spectroscopy**

FTIR measurements occurred as described before [[32](#_ENREF_32)]. Briefly, the isolated SC was placed between two AgBr cells and put under continuous dry air for 30 min before the start of measurements. All spectra were acquired on a Varian 670-IR spectrometer equipped with a broad-band mercury cadmium telluride detector. The spectrometer was cooled with liquid nitrogen and connected to a controlled heating device. The spectrometer collected the data with a frequency range of 400-4000 cm^-1^. The spectral parameters were speed 25 kHz, UDR 2, filter 6.4 and aperture 1 cm^-1^. The measurements were performed with a 240 seconds time resolution and a length of run equal to 164 min. The lateral packing behaviour was examined between 0°C and 40°C with a heating rate of 0.25°C/min, resulting in a 1°C temperature increase per measurement. FTIR spectra were analysed and deconvoluted with Varian Resolutions Pro software and finally processed with a custom designed XRAY plot program.

# Supplementary table

**Supplementary table 1.** Antibody specifications which are used during immunohistochemistry and immunofluorescence analyses.

| **Immunohistochemistry** | **Material** | **Origin** | **Clone** | **Dilution** | **2^nd^ Ab** | **Manufacturer** |
| --- | --- | --- | --- | --- | --- | --- |
| ***Primary antibody*** | | | | | | |
| Ki67 | FFPE | Mouse | MIB1 | 1:100 | A | DAKO, Denmark |
| Cytokeratin 10 | FFPE | Mouse | DE-K10 | 1:50 | A | Labvision/Neomarker, USA |
| Involucrin | FFPE | Mouse | SY5 | 1:1200 | A | Sanbio, The Netherlands |
| Cytokeratin 16 | FFPE | Mouse | LL025 | 1:100 | A | Serotec, UK |
| Cytokeratin 17 | FFPE | Mouse | E3 | 1:1500 | A | EMD Millipore Corporation, USA |
| ***Secondary antibody*** | | | | | | |
| A) Biotinylated goat  anti-mouse |  | Goat |  | 1:200 |  | Southern Biotechnology, USA |
| **Immunofluorescence** |  | **Origin** | **Clone** | **Dilution** | **2^nd^ Ab** | **Manufacturer** |
| ***Primary antibody*** | | | | | | |
| Loricrin | FFPE | Rabbit | AF62 | 1:1000 | B | Covance, The Netherlands |
| Filaggrin | FFPE | Rabbit | PRB417 | 1:1000 | B | Covance, The Netherlands |
| Collagen type IV | FFPE | Mouse | 24.12.8 (PHM-12) | 1:75 | C | Chemicon, Australia |
| Vimentin | FFPE | Mouse | V9 | 1:250 | C | Sigma-Aldrich, Germany |
| GBA | FFPE | Rabbit | EPR5142 | 1:150 | B | Abcam, UK |
| aSMASE | FFPE | Rabbit | polyclonal | 1:1000 | B | Abcam, UK |
| ***Secondary antibody*** | | | | | | |
| B) Cy3-conjugated anti-rabbit |  | Goat |  | 1:500 |  | Jackson immunoresearch Laboratory, USA |
| C) Cy3-conjugated anti-mouse |  | Goat |  | 1:1000 |  | Jackson immunoresearch  Laboratory, USA |

**Supplementary figures**


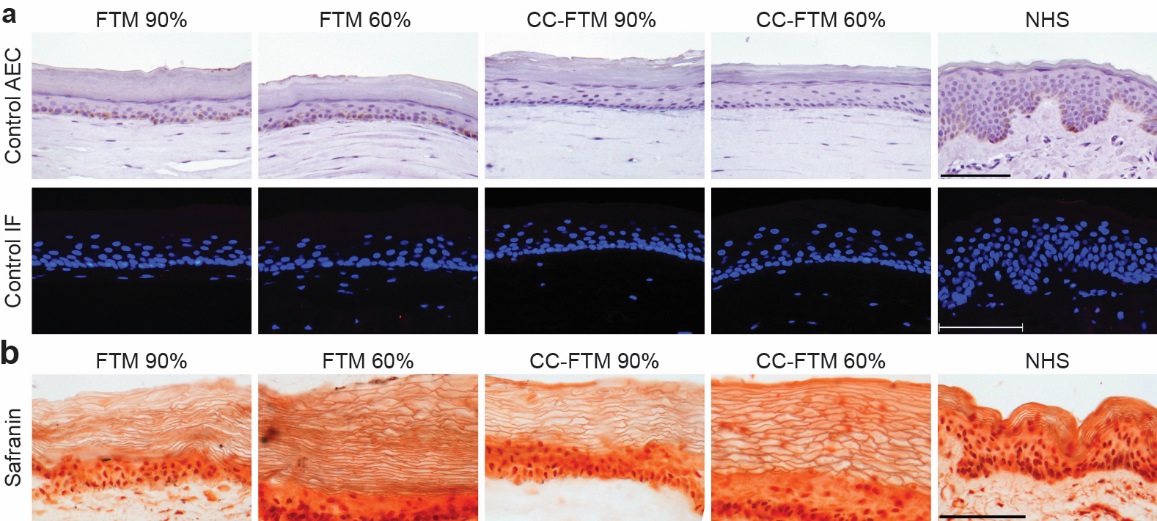


**Supplementary figure S1.** **Epidermal morphogenesis protein biomarker controls and stratum corneum thickness analysis. (a)** Immunohistochemical control sections for the 3-Amino-9-ethylcarbazole (AEC) method and immunofluorescence (IF) method. **(b)** Safranin red stained lipid matrix of the stratum corneum imaged after alkali expansion of the corneocyte layers. Scale bar indicates 100μm.


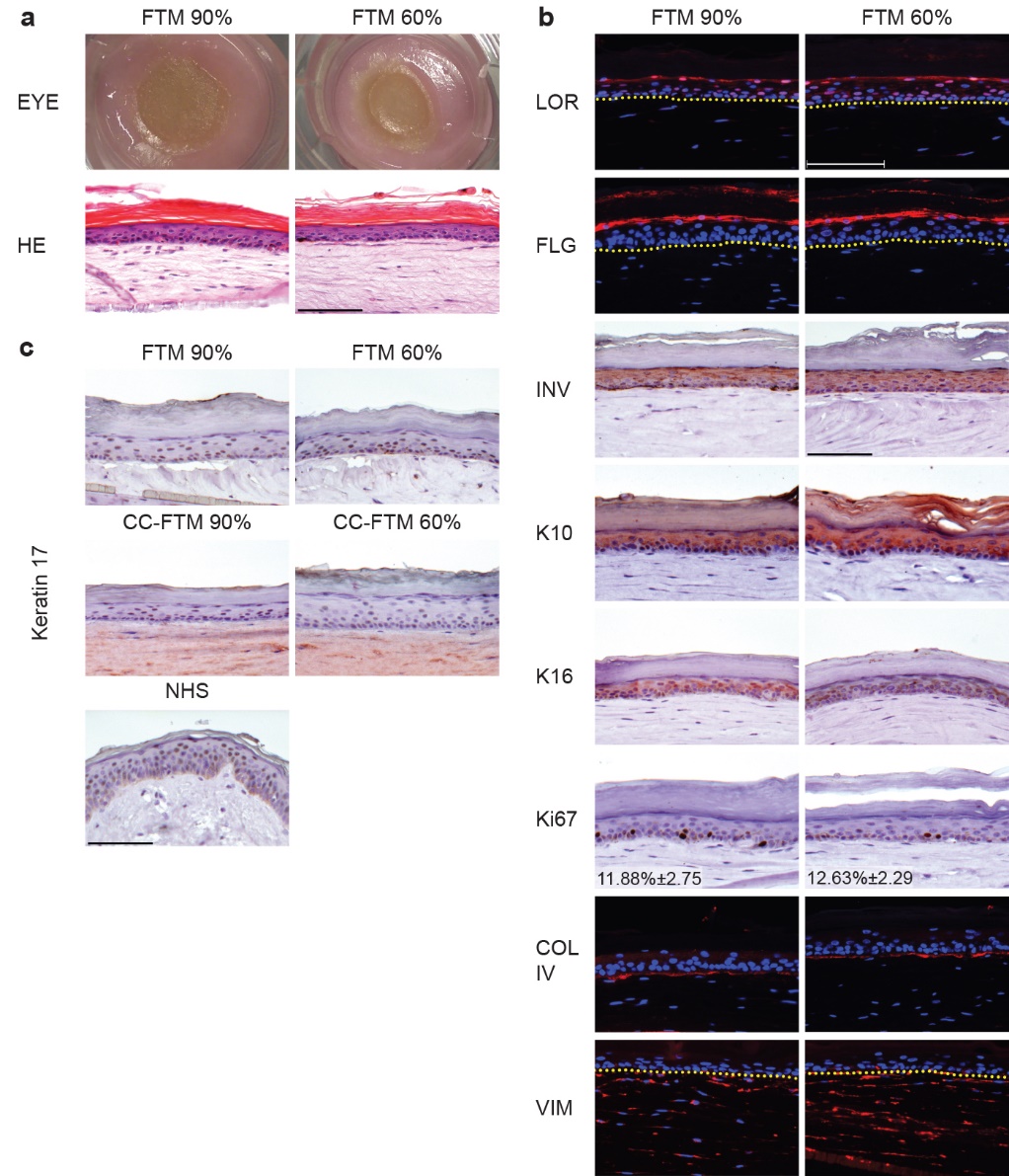


**Supplementary figure S2.** **Generation of full thickness models at reduced relative humidity.** (**a**) General morphology in high (90%) and low (60%) RH, assessed by eye and HE staining. **(b)** Expression of loricrin (LOR), filaggrin (FLG), involucrin (INV), keratin 10 (K10), keratin 16 (K16) indicating epidermal differentiation programs and activation respectively. Proliferation was assessed by Ki67 (with indicated proliferation index as mean ± SD, N=4), basement membrane by collagen type IV (COL IV), and fibroblast distribution by vimentin (VIM). **(c)** Keratin 17 expression in HSEs and in NHS. Protein biomarkers are shown in red and nuclei in blue. Yellow dotted line indicates dermal-epidermal junction. Scale bar indicates 100μm.


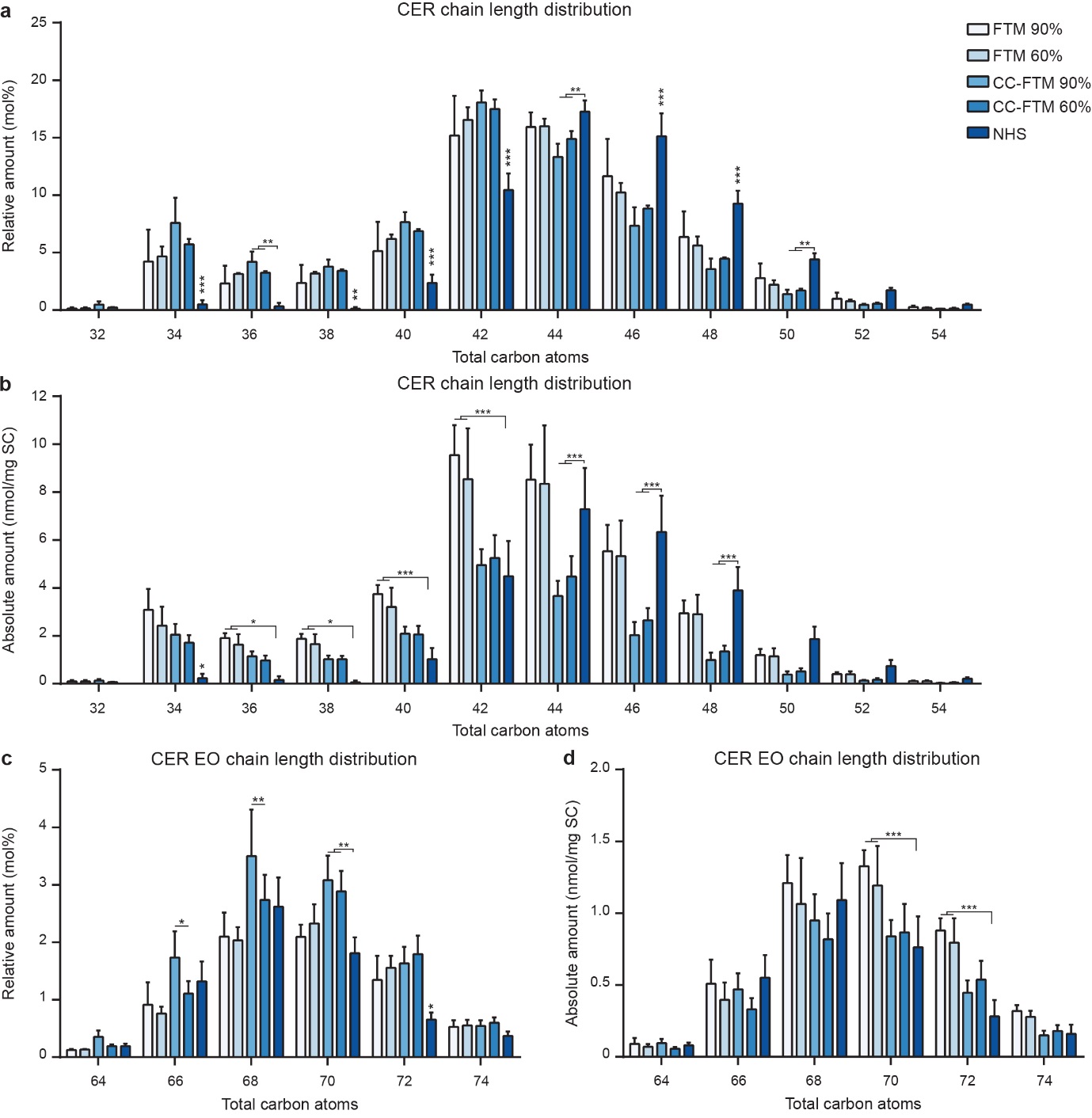


**Supplementary figure S3.** **Carbon chain length distribution of CERs and CERs EO.** Bar plot showing the carbon chain length distributions of even numbered CER between C32 and C54 and of even numbered CERs EO between C64 and C74. Data is presented in relative amount **(a, c)** and in absolute amount per mg stratum corneum **(b, d).** Data is shown for both HSE types developed at 90% and 60% RH and of NHS. Data represents mean + SD, N=4.


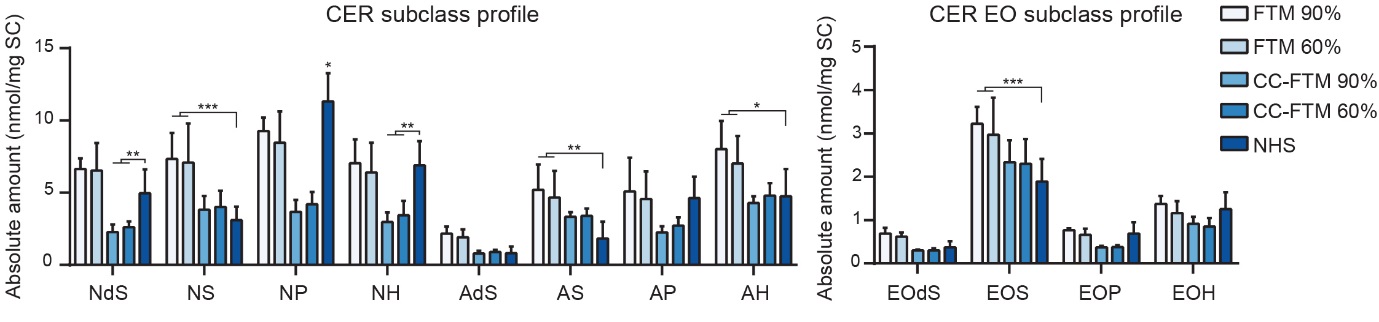


**Supplementary figure S4.** **Quantitative CER and CER EO subclass profile.** Bar plot showing the CER and CER EO subclass profiles as absolute amounts per mg stratum corneum. Data is shown for both HSE types developed at 90% or 60% RH and for NHS, represented by mean + SD, N=4.

**
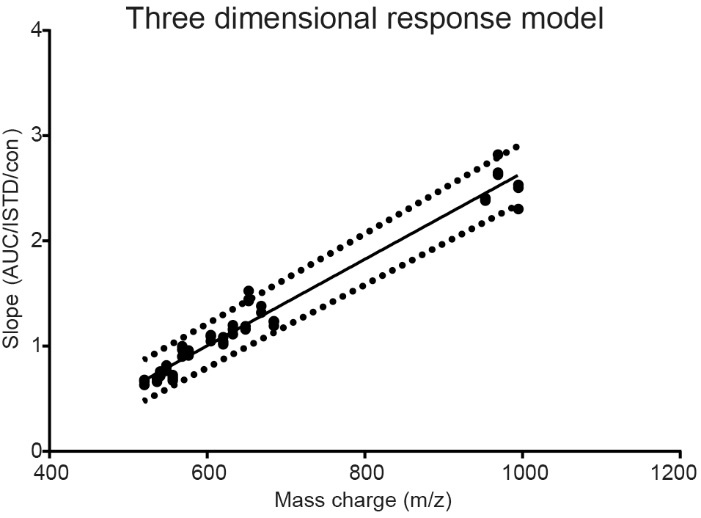
**

**Supplementary figure S5.** **Three-dimensional response model.** The model is generated based on settings of the mass spectrometer, compound properties, and a limited number of synthetic ceramides according to Boiten *et al.* [[2](#_ENREF_2)].
